# A Fibre-Optic Platform for Sensing Nitrate Using Conducting Polymers

**DOI:** 10.3390/s21010138

**Published:** 2020-12-28

**Authors:** Soroush Shahnia, Heike Ebendorff-Heidepriem, Drew Evans, Shahraam Afshar

**Affiliations:** 1 Laser Physics and Photonic Devices Laboratories, UniSA STEM, University of South Australia, Mawson Lakes, SA 5095, Australia; Shahraam.AfsharVahid@unisa.edu.au; 2 ARC Centre of Excellence for Nanoscale Biophotonics, Institute for Photonics and Advanced Sensing, The University of Adelaide, Adelaide, SA 5000, Australia; heike.ebendorff@adelaide.edu.au; 3 Future Industries Institute, University of South Australia, Mawson Lakes, SA 5095, Australia

**Keywords:** nitrate sensing, optical fibre, PEDOT, conducting polymer

## Abstract

Monitoring nitrate ions is essential in agriculture, food industry, health sector and aquatic ecosystem. We show that a conducting polymer, poly(3,4-ethylenedioxythiophene) (PEDOT), can be used for nitrate sensing through a process in which nitrate ion uptake leads to oxidation of PEDOT and change of its optical properties. In this study, a new platform is developed in which a single-mode fibre coated at the tip with PEDOT is used for nitrate sensing. A crucial step towards this goal is introduction of carbonate exposure to chemically reduced PEDOT to a baseline value. The proposed platform exhibits the change in optical behaviour of the PEDOT layer at the tip of the fibre as it undergoes chemical oxidation and reduction (redox). The change in optical properties due to redox switching varies with the intensity of light back reflected by the fibre coated with PEDOT. The proposed platform during oxidation demonstrates linear response for the uptake of nitrate ions in concentrations ranging between 0.2 and 40 parts per million (ppm), with a regression coefficient $R^{2}=0.97$ and a detection limit of 6.7 ppm. The procedure for redox switching is repeatable as the back reflection light intensity reaches ±1.5% of the initial value after reduction.

## 1. Introduction

Ions play an important role in many biological and engineering processes. Their concentration in aqueous solution is important for practical applications such as defining the quality of drinking water, the treatment of wastewater and production of food [1,2]. Nitrate is an ion of interest in several of these scenarios; for example, it plays a key role in the nitrogen cycle in agriculture [3], removing the hazardous contaminants from the aquatic environment [4,5,6], and in measuring nitrate oxide in blood [7]. Quantifying the concentration of nitrate in water is critical to ensure an efficient use of the ions while avoiding any potentially harm impact caused by overuse in environment. Excess nitrate (NO${}_{3}^{-}$) from agriculture can ultimately find its way into drinking water due to high water solubility of NO${}_{3}^{-}$ and be ingested by humans. When in vivo, NO${}_{3}^{-}$ can be spontaneously reduced to nitrite (NO${}_{2}^{-}$), which interferes with the oxygen transport mechanism within the body by the irreversible conversion of haemoglobin to methaemoglobin in red blood cells [8]. To combat this, the Environmental Protection Agency (EPA) has determined the safe levels of nitrogen in potable water to be 10 parts per million (ppm or 10 mg/L) equivalent to 44 ppm of NO${}_{3}^{-}$ [9,10]. Examples such as this serve as motivation for the specific sensing of NO${}_{3}^{-}$.

NO${}_{3}^{-}$ can be detected through various scientific methods including spectroscopic [11,12], electrochemical [13], chromatography [14,15] and capillary electrophoresis [16]. One of the main accepted detection methods of nitrogen in laboratories is Kjeldahl digestion [17,18]. This method is considered as the reference way of detecting nitrogen in biological samples such as meat, plants and wastewater [19]. In this method, the sample preparation and procedure contains several steps and the detection of other forms of nitrogen containing compounds such as NO${}_{3}^{-}$ and NO${}_{2}^{-}$ is not possible without an extra step of reduction to ammonium. Another accepted method by laboratories and scientists is colorimetric detection of NO${}_{3}^{-}$ by a complex reagent [20,21,22]. The complex reagent is then reduced to form a coloured compound and the NO${}_{3}^{-}$ level can be determined by spectroscopy in the visible spectrum. Despite the fact that colorimetric- and spectroscopic-based techniques are highly sensitive (detection levels of only a few ppm), most of them have several sample preparation steps which are time consuming and in many cases hard to apply in the field. Another popular technique is using NO${}_{3}^{-}$ ion-selective electrodes for deployment in water. The electrochemical performance analysis of NO${}_{3}^{-}$ electrode has a disadvantage of selectivity. The electrodes typically respond to ions according to Hofmeister selectivity series [23,24]. Recently, the selective uptake of NO${}_{3}^{-}$ from soil pore water has been demonstrated independent of the Hofmeister selectivity using a thin film coating of poly(3,4-ethylenedioxythiophene)(PEDOT) in an electrochemical process [25]. In a separate study, this conducting polymer material was deposited onto the end of an optical fibre as a step towards a new optical sensor [26].

Herein, the NO${}_{3}^{-}$ concentration of the order of a few ppm was detected in Milli-Q water using an optical method. An optical fibre coated with PEDOT at its tip is used as the optical sensor. The idea is to take advantage of the small fibre area of exposure and the uptake of NO${}_{3}^{-}$ by PEDOT and monitor the difference in the back reflection in real-time. In contrast to existing NO${}_{3}^{-}$ concentration sensing methods with PEDOT [25,27], the current approach does not rely on measuring the potential difference, but rather uses the optical back reflection as a sensing mechanism. Conducting polymers, and especially PEDOT, are well reported to undergo doping and de-doping by inserting and removing anions from within. This reversible process is accompanied by changes in the optical properties of the PEDOT and typically exploited in electrochromic devices [28,29,30]. For the sensing device reported herein, NO${}_{3}^{-}$ is used as the dopant, with a basic solution (pH ≈ 10.5) of 1 mM CO${}_{3}^{2-}$ used to chemically reduce the PEDOT [31] (similar to hydroxide [32] and polyamide solutions [33]) and thus resetting the sensing probe. The PEDOT doped with the Tosylate anion (PEDOT:Tos) is used to investigate oxidation and reduction (redox) switching in Milli-Q water. The measurement is performed in real-time by monitoring the changes in back reflection from the PEDOT:Tos coated at the tip of the fibre. This study is based on optical reflection at a single wavelength (1550 nm). PEDOT spectra showed higher absorption at longer wavelengths, with the transition of polaron and bipolaron states of charged carriers to a higher order of electron paired and unpaired states that results in a local minima at around 1100 nm [26,34]. This results in a better electro-optical switching at 1550 nm in comparison to visible region and shorter wavelengths [35]. Therefore, a stable and narrow linewidth laser at the telecom wavelength of 1550 nm is a suitable source for this study. Wide availability of fibre-pigtail lasers at this wavelength allows us to have a compact, all-fibre system with no need for optical alignment, thus avoiding power fluctuations. The intrinsic benefits of optical fibres coated with PEDOT, i.e., biocompatibility, small cross section, remote interrogation and widespread communications infrastructure, suggest that the using optical fibre in combination with PEDOT can be considered as a forcible platform for NO${}_{3}^{-}$ sensing in the future. Both PEDOT and optical fibres, being in vivo friendly [36,37,38,39], have a significant edge over other NO${}_{3}^{-}$ sensing techniques, and the detection of NO${}_{3}^{-}$ in living organisms is often required in agriculture and health sector. However, the challenge of consistent fibre-PEDOT fabrication and preventing sample contamination still have to be overcome. This work is an initial step in using optical fibre coated with PEDOT for sensing applications.

## 2. Materials and Methods

**Figure 1:**
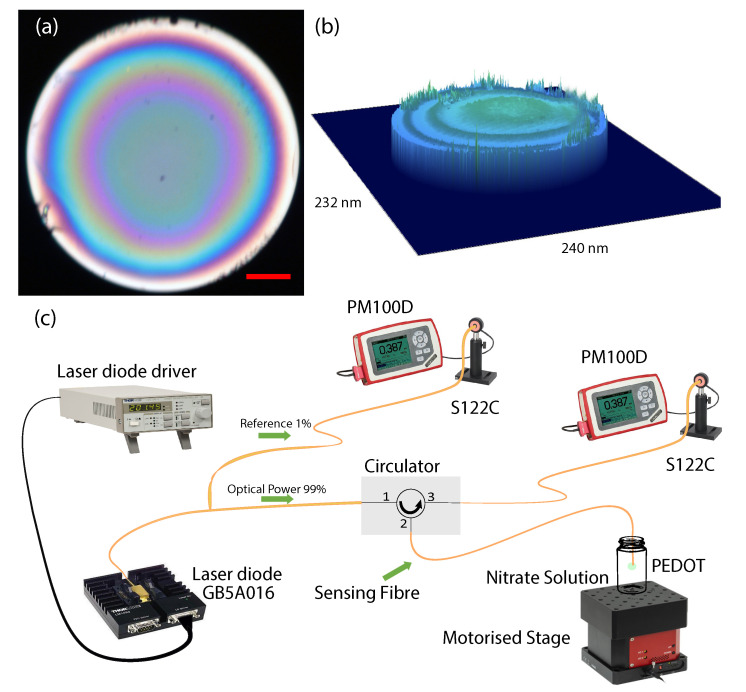
(**a**) Cross-sectional image on reflection mode of PEDOT at the tip of a SMF-28 fibre. Scale bar represent 20 μm (red line), (**b**) 3D surface profile of PEDOT at the tip of the fibre using confocal profilometry and (**c**) experimental setup for measuring NO${}_{3}^{-}$ concentration based on optical back reflection.

The sensing probe consists of a PEDT:Tos layer at the tip of an optical fibre (Figure 1a). The PEDOT:Tos composite was fabricated through a vapour phase polymerization according to a previously published protocol [26]. In summary, this technique involves the following four main steps: (1) Preparation of the oxidant solution. (2) Deposition of an oxidant layer at the tip of the fibre. (3) The fibre coated with oxidant is placed inside a vacuum chamber where it is exposed to monomer vapour. (4) Post-deposition treatment of the PEDOT layer by dipping the tip of the fibre into ethanol to remove any unreacted monomers, oxidants and other by-products. Prior to vapour phase polymerization, an oxidant solution was prepared. The oxidant solutions contain Fe(III) tosylate, which was received from H. C. Starck as a 54 wt.% solution in butanol (Baytron CB 54). Sodium carbonate (Na${}_{2}$CO${}_{3}$), sodium nitrate (NaNO${}_{3}$), 3,4-ethylenedi-oxythiophene (EDOT) monomer and the block copolymers poly(ethylene glycol-propylene glycol-ethylene glycol) (PEG-PPG-PEG) (Mw = 5800 Da; trade name = P123) were obtained from Sigma-Aldrich. All chemicals were used without further purification. Various solutions of NaNO${}_{3}$ were prepared in Milli-Q water at different concentrations. A stock solution of 200 ppm (3.23 mM) NO${}_{3}^{-}$ was prepared. A subsample of this solution was taken and diluted with Milli-Q water to reach 100 ppm NO${}_{3}^{-}$. This process of subsampling and diluting was repeated for each new concentration until a series of NO${}_{3}^{-}$ solutions were prepared: 0.2, 2, 5, 10, 20, 30, 40 and 50 of NO${}_{3}^{-}$. The 1 mM Na${}_{2}$CO${}_{3}$ solution is used as a basic solution, with a measured pH of 10.5. SMF-28-100 optical fibres having a core diameter of 8.2 μm and a cladding diameter of 125 ± 0.7 μm were obtained from Thorlabs Inc. A Cary 5000 UV–Vis NIR spectrophotometer (Agilent Technologies, CA, USA) was used to conduct the spectroscopy of the PEDOT samples deposited on a soda-lime-silica glass (refractive index, *n* = 1.5 at wavelength 1550 nm) substrate before and after exposure to NO${}_{3}^{-}$ and CO${}_{3}^{2-}$ solutions.

Figure 1c illustrates the optical setup for sensing. The light source was a linearly polarized single mode 1550 nm continuous wavelength laser diode beam (Thorlabs DJ532-40, GN5A016). The beam propagates through a coupler; 1% of the optical power measured by a Thorlabs S122C photodiode to probe the stability of the laser during the experiment and 99% of the optical power propagates from terminal 1 to 2 of the circulator. Prior to the experiments, the optical power was set at 2 mW at terminal 2 of the circulator (below the optical damage threshold of PEDOT [26]). The diode laser’s output was stable with <0.2% optical power fluctuation over the duration of the experiments. The terminal 2 of the circulator is connected to the input of the sensing fibre. The output end of the sensing fibre was flat-cleaved (≤1${}^{\circ }$) and coated with the PEDOT:Tos. The coating process has been explained in detail in our previous work [26]. In summary, an oxidizer solution was dip-coated onto the end of the fibre, and subsequently the vapour phase polymerization technique [28] was employed to yield 200–250 nm thick PEDOT:Tos at the fibre end (see Figure 1b). The fibre coated with PEDOT was held stationary to remove any bending loss and/or power fluctuations before dipping into the solution. The solution was centred on a motorized stage and the motor was programmed to bring the solution up and down as the tip of the fibre was dipped into the solution. The light back reflected was passed through terminal 2 to 3 of the circulator and was measured by a Thorlabs S122C photodiode. The difference in back reflected light intensity before and after dipping the sensor in the nitrate solution was used to measure the concentration of NO${}_{3}^{-}$.

## 3. Results and Discussion

**Figure 2:**
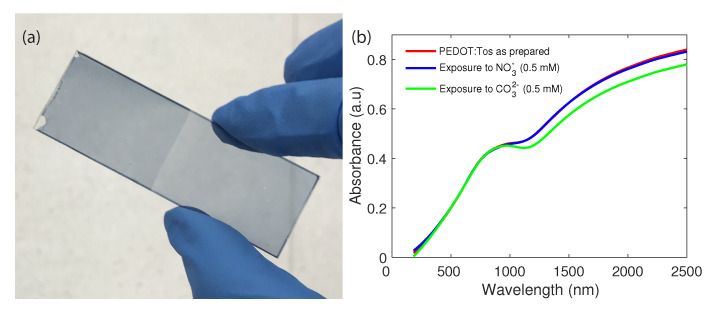
(**a**) The change in PEDOT color from light blue to dark blue in the visible due to oxidation by NO${}_{3}^{-}$ (1 mM) after 24 h exposure, (**b**) absorbance spectra of PEDOT before and after exposure to NO${}_{3}^{-}$ and CO${}_{3}^{2-}$ for 2 min.

The spectral response of PEDOT before and after exposure to NO${}_{3}^{-}$ and CO${}_{3}^{2-}$ has been investigated. In the visible range, the PEDOT layer changes from light blue (as-deposited PEDOT) to a darker blue (after NO${}_{3}^{-}$ exposure) (see Figure 2a). As the same PEDOT sample is exposed to CO${}_{3}^{2-}$, it switches to an even darker blue appearance, although the spectral difference is minuscule. The visible change in colour of the PEDOT layer is an indication of the polymers interaction with the surrounding aqueous solution [31]. Figure 2b shows absorbance spectrum (400–2500 nm) of a thin layer (200 nm) of PEDOT prepared on the soda-lime silica glass before and after exposure to NO${}_{3}^{-}$ and CO${}_{3}^{2-}$ for 2 min. As-deposited PEDOT is highly oxidised, and thus there is little change in the absorption spectra after exposure to NO${}_{3}^{-}$. However, there is a decrease in absorption after exposure to CO${}_{3}^{2-}$ due to chemical reduction from the basic solution (pH >7), in line with previous studies on the pH response of PEDOT [32]. Rather than delve into the mechanism of chemical oxidation and reduction, herein the resultant change in PEDOT optical properties in the short-wave infrared (SWIR) region will be exploited for sensing NO${}_{3}^{-}$. This SWIR response conveniently overlaps with the commonly used optical wavelength of 1550 nm (as highlighted in previous studies [26]).

**Figure 3:**
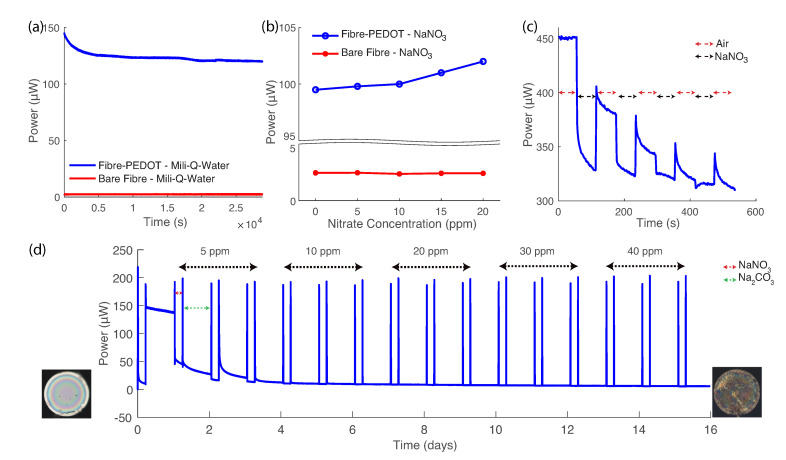
Properties of PEDOT before and after exposure to NO${}_{3}^{-}$. (**a**) PEDOT back reflection by reduction in Milli-Q water (blue) and back reflection of fibre (red), (**b**) response of both fibres after addition of droplets of NO${}_{3}^{-}$ into Milli-Q water, (**c**) a PEDOT sample exposed repeatedly to cycles of NO${}_{3}^{-}$ solution (50 ppm) and air and (**d**) reusability testing: repeated cycles of NaNO${}_{3}$ and Na${}_{2}$CO${}_{3}$ with 2 min of air in between. The image on the left is the cross section image of PEDOT at the tip of the fibre in reflection mode before the experiment and the one on the right is after all the exposure to NO${}_{3}^{-}$ and CO${}_{3}^{2-}$ for 21 days.

A different set of experiments was designed to understand the reaction of PEDOT with NO${}_{3}^{-}$ in Milli-Q water (see Figure 1c). Figure 3a shows the results for two SMF-28 fibres dipped into Milli-Q water: one with PEDOT at the tip, the other a bare flat-cleaved fibre. The tip of both fibres were dipped into a vial containing 15 ml of liquid for a period of 12 h. The magnitude of the back reflection is significantly different when the PEDOT coating is present. The change in back reflection with time is hypothesised to be related to changes in the PEDOT layer thickness, most likely due to the presence of hygroscopic non-ionic triblock copolymer chains (used to template the growth of the PEDOT during the deposition [40]). Long-term soaking of PEDOT in water has been previously reported for the creation of batteries and high humidity sensors [41,42]. After the soaking period, a stock solution of NO${}_{3}^{-}$ was added dropwise to the vial, increasing its concentration in solution. The increase in back reflection observed for the PEDOT relates to its changing oxidation level as the NO${}_{3}^{-}$ concentration changes, in contrast to the constant response of the bare fibre (Figure 3b). The constant response for the bare fibre is in agreement with literature studying low concentrations (<1000 ppm) where the change in the solution’s refractive index is negligible [43,44]. The change in index of refraction of water is also dependent on temperature and pressure in addition to the NO${}_{3}^{-}$ concentration [45]. The pressure and temperature in this study are considered constant; atmospheric pressure and the temperature in the optical laboratory are monitored and set at 101.325 kPa and 22 ${}^{\circ }$C, respectively. The results in Figure 3b indicate that the change in the back reflection of PEDOT is due to its oxidation level, not changes in the refractive index of the medium.

As a result of the changing PEDOT oxidation level, the refractive index of the layer also changes, providing a means to optically measure PEDOT’s interaction with the surrounding medium. However, when PEDOT is repeatedly exposed to a NO${}_{3}^{-}$ solution the optical response plateaus (Figure 3c). This plateau occurs when an equilibrium oxidation level is reached for the given NO${}_{3}^{-}$ concentration being tested/sensed. For example, Figure 3c represents the results where the PEDOT was exposed repeatedly to cycles of 1 min in 50 ppm NO${}_{3}^{-}$ solution following 1 min in air. The ultimate saturation of the optical response indicates that an additional step is required to realise utility of this sensing platform. The additional step involves exposure to a solution that can reset the oxidation level of PEDOT to a consistent value (and relatively lower compared to those achieved upon NO${}_{3}^{-}$ exposure).

To analyse the optical response of PEDOT at different NO${}_{3}^{-}$ concentration, a basic solution was employed (pH >7). Na${}_{2}$CO${}_{3}$ was used in solution at low concentration to achieve a pH of ∼10.5, in order to chemically reduce the PEDOT to a lower oxidation level. The details of this mechanism and its reversible nature were recently described by Sethumadahavan et al. [31]. In comparison to commonly used NaOH and polyamide solutions, the Na${}_{2}$CO${}_{3}$ solution is envisaged to lower the oxidation level of PEDOT without inflicting irreversible damage associated with strongly basic solutions [32]. The change in back reflection upon CO${}_{3}^{2-}$ exposure is correlated with the dynamics of water reacting with the positive charges on PEDOT, causing the liberation of NO${}_{3}^{-}$. Figure 3d illustrates the back reflection response of PEDOT at 1550 nm to repeated exposure to NaNO${}_{3}$ and Na${}_{2}$CO${}_{3}$ solutions for a range of increasing NO${}_{3}^{-}$ concentrations (5 to 40 ppm). The sharp peaks in back reflection represent points in time where the fibre is removed from solution to the lab air environment, prior to submersion in the next solution. These peak values correlate well with the concentration of NO${}_{3}^{-}$ in solution. The before and after exposure microscope images in reflection mode are included in Figure 3d to illustrate what visually happens to PEDOT at the tip of the fibre. PEDOT was initially exposed to NO${}_{3}^{-}$ for 5 h, then it was dried in air for 2 min. Afterwards, PEDOT:NO${}_{3}^{-}$ was dipped into the CO${}_{3}^{2-}$ solution for 19 h. The long exposure times is to ensure all the NO${}_{3}^{-}$ ions are removed from the sample before re-exposing. The sample was dipped into a single NO${}_{3}^{-}$ concentration 3 times over 3 consecutive days, before proceeding to the next concentration. The back reflection in air (after dip in solution) was compared between the given NO${}_{3}^{-}$ concentration and the reference CO${}_{3}^{2-}$ solution. The deviation in the response characteristics in back reflection upon exposure to CO${}_{3}^{2-}$ was found to be ±1.5%, highlighting the usability of CO${}_{3}^{2-}$ for PEDOT as a reference point in NO${}_{3}^{-}$ sensing measurements.

**Figure 4:**
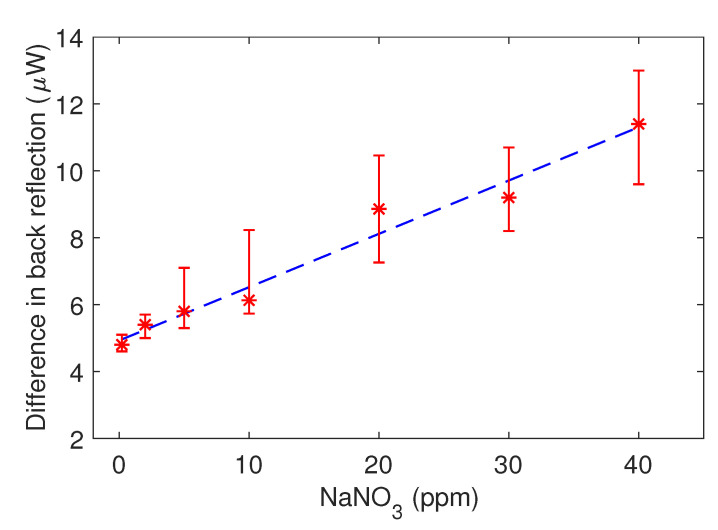
Characterization of the sensor. The difference in back reflection power from the PEDOT layer before and after exposure to nitrate is plotted against the NaNO${}_{3}^{-}$ concentration. The red stars with error bars represent the experimental data and the blue dashed line represents the linear regression fit with a coefficient of determination of 0.97.

Figure 4 illustrates the difference in back reflection (CO${}_{3}^{2-}$ - NO${}_{3}^{-}$) response as a function of NO${}_{3}^{-}$ concentration. The linear fit shown in Figure 4 produced a regression coefficient of 0.97 with the slope of 0.16±0.03μW ppm${}^{-1}$ for NO${}_{3}^{-}$ concentrations between 0.2 and 40 ppm. The error bar were calculated by repeating the experiments three times consecutively. The results shows reusability with 21 measurement with one PEDOT sample. The results also allowed for retrieving the limit of detection (LOD), 6.7 ppm, which is defined as LOD = 3σ, with σ = 2.23 being the standard deviation derived from 3 different sample with each one contaminated by NO${}_{3}^{-}$ and CO${}_{3}^{2-}$ 21 times for a total number of 63 values.

**Figure 5:**
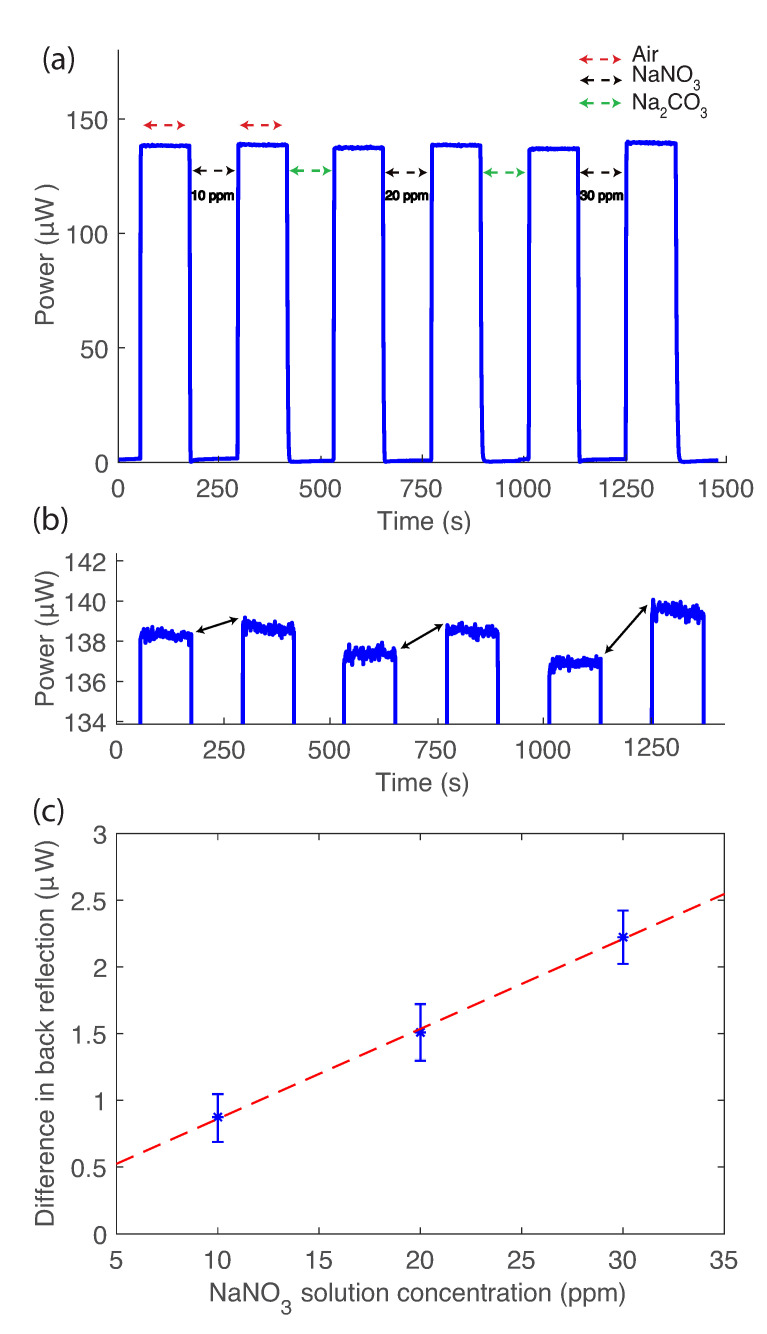
Response time of the sensor. (**a**) Three cycles of NaNO${}_{3}$ and Na${}_{2}$CO${}_{3}$ with 2 min in solution and 2 min in air for different NO${}_{3}^{-}$ concentration of 10, 20 and 30 ppm, respectively, (**b**) zoomed in section of part a, the black double arrow shows the difference in back reflection before and after exposure to NaNO${}_{3}$, and (**c**) the difference in back reflection light intensity is plotted against the NaNO${}_{3}^{-}$ concentration. The blue stars with error bars represent the experimental data and the red dashed line represents the linear regression fit.

Another important parameter for the sensor characterisation is its response time. Herein, the response time is defined as the exposure time to a particular analyte that is required to achieve a stable back reflection for two consecutive measurements in air after the fibre probe comes out of a particular analyte concentration, while in the above experiments, to make sure that the probe has been fully reset, we immersed the probe in CO${}_{3}^{2-}$ after each NO${}_{3}^{-}$ exposure for 19 h. In order to see if it would be possible to have a shorten exposure time preliminary series of experiments were conducted. it appears that an initial step of 19 h exposure to CO${}_{3}^{2-}$ to remove most of the tosylate [31] followed by a 2 min consecutive exposure to NO${}_{3}^{-}$ is sufficient to reset the probe to the same value of back reflection (±1.5%). In all measurements, initially fibre probe coated with PEDOT dipped into CO${}_{3}^{2-}$ solution (1 mM) for 19 h as a “resetting” step. In all consecutive steps, the CO${}_{3}^{2-}$ exposure time was reduced to 2 min, as shown in Figure 5a. The probe back reflection has been reset close to its initial value (±1.5%) after each CO${}_{3}^{2-}$ exposure which is in agreement with our previous measurements (Figure 3c). After the fibre probe comes out of the Na${}_{2}$CO${}_{3}$ and exposed to air for 2 min, it takes less than 4 s for the response time in air to reach the plateau state. Figure 5b, shows the zoomed in results from part Figure 5a for 3 different NaNO${}_{3}$ concentration measurements: 10, 20 and 30 ppm. The time that considered in this experiments is 2 min between each step. This time is required to change the solutions in each step. To check the stability of the sensor, we repeat the procedure 3 times with the same fibre probe and hence the error bar shown in Figure 5b. In this sample, due to lower back reflection relative to the sample in Figure 4, the slope of the fitted line (0.09±0.02μW ppm${}^{-1}$) is less than our previous measurement. We believe that the main reason for lower back reflection is the variation in sample thickness of ±50 nm and cleave angle of $\pm 1^{\circ }$. However, if we linearly correlated the amount of back reflection to the same level as back reflection as Figure 3c, the slope of the line will be 0.123±0.03μW ppm${}^{-1}$. The correlated slope is in the range of error and in agreement with our previous results. Therefore, the 2-min interval was considered as the response time.

The conductive polymer PEDOT is observed to repeatedly oxidise with NO${}_{3}^{-}$ and reduce with CO${}_{3}^{2-}$. The change in intensity of back reflection observed for NO${}_{3}^{-}$ concentrations as low as 0.2 ppm. Such sensitivity is the point of interest in relation to drinking water, wastewater and water in agricultural soils where NO${}_{3}^{-}$ is present. The sensor platform surpasses the requirement of measuring NO${}_{3}^{-}$ concentration below 44 ppm in drinking water by one order of magnitude. Compared to the previously reported work of NO${}_{3}^{-}$ sensing using PEDOT [25,27], this research employed an optical sensing method rather than an electrochemical one. The current method shows reusability through the ability to “reset” the sensor and this characteristic can be subsequently used for calibration and real-time monitoring. This work provides an alternative approach to some of the conventional and sophisticated NO${}_{3}^{-}$ sensing methods [17,18,19,20,21]. For example, ion chromatography, one of the most commonly used and accepted techniques, can measure as low as 0.05 ppm but requires an expensive bulky equipment [14,15]. Ramaswami et al. compared a range of techniques to measure NO${}_{3}^{-}$ in the presence of high concentrations of Cl${}^{-}$ [46]. The reported minimum measurable concentration across those studies was 0.2 and 0.02 ppm. Parveen et al. coated a section of large core fibre (600 μm) with CNTs/Cu-NPs nanocomposite and measured the shift in surface plasma resonance frequency in the visible region of spectrum [47]. They achieved the best value in limit of detection described in the literature, i.e., 0.004 ppm. However, the main issue with surface plasma resonance devices is repeatability. Losses in optical power and alterations in optical coupling drastically changes the performance of such sensors [48]. On top of that, moving the fibre will cause a phase shift, which makes it impractical for applying in the field. In a separate study, an etched fibre Bragg grating was used to measure the NO${}_{3}^{-}$ by monitoring the shift in the reflected wavelength with a limit of detection of 3 ppm [49]. Fibre-optic sensors offer several advantages over chemical, electrochemical and electric transducers with regards to their small size, in situ/vivo friendly characteristics and the possibility of being deployed as distributed sensors. Here, we developed and studied a chemosensor that has the ability to take advantage of both chemical and optical parameters. From the practical aspect the PEDOT-coated optical fibre herein is easily fabricated and operated, and may overcome some of the practical challenges to allow in-field measurement. Future work will focus on using this platform for the analysis of NO${}_{3}^{-}$ and NO${}_{2}^{-}$ compounds in water environments, with particular focus on the ultimate measurement sensitivity, and the detectable concentration range.

## 4. Conclusions

To conclude, a fibre-optic platform utilising PEDOT at the tip of the fibre has been fabricated and characterised. In this platform, the NO${}_{3}^{-}$ sensing mechanism relies on redox switching capability of PEDOT. The PEDOT chemically oxidised in the presence of NO${}_{3}^{-}$ after being reduced within ±1.5% of its initial value in the presence of CO${}_{3}^{2-}$. The proposed platform during oxidation demonstrates linear response with the slope of 0.16±0.03μW ppm${}^{-1}$ for the uptake of NO${}_{3}^{-}$ in the concentration range of 0.2 to 40 ppm, with a regression coefficient $R^{2}=0.97$ and a limit of detection of 6.7 ppm. The response time of PEDOT has been measured to be 2 min with the ability of reaching plateau state and recovery in less than 4 s. This new sensing platform is configured as a fibre probe, with benefits such as capability of remote sensing, better area of selection due to a small diameter (125 μm) and less intrusiveness for in vivo measurements. In the future, a fibre-based distributed sensor coated with PEDOT can monitor real-time information on NO${}_{3}^{-}$ concentrations in a large area such as seawater where collecting discrete samples found to be insufficient for providing statistical significance. Further investigation is in progress to enhance the reproducibility and long term stability of PEDOT, with an ultimate goal of coating on the side of an optical fibre for constructing a distributed optical sensor.

## Data Availability

The data presented in this study are available on request from the corresponding author. The data are no publicly available as they involve the subsequent application of patent for invention and the publication of project deliverables.
